# Maintaining Paediatric Resuscitation Readiness: A Clinical Teacher's Toolbox for Low‐Dose, High‐Frequency Practice

**DOI:** 10.1111/tct.70517

**Published:** 2026-08-31

**Authors:** Weerapong Lilitwat

**Affiliations:** ^1^ Department of Pediatrics Texas Tech University Health Sciences Center School of Medicine Lubbock Texas USA

**Keywords:** clinical teaching, distributed practice, feedback, interprofessional education, paediatric resuscitation, simulation, skills retention

## 1 Introduction

A clinician may arrive on a paediatric service with current life‐support certification and still feel uncertain when asked to perform under pressure. Certification remains important. It confirms that the learner completed a recognised course and met its standard at that time. It does not, by itself, show that compressions, ventilation, leadership, role allocation and communication remain readily available months later in a crowded clinical environment. Resuscitation skills can decay, particularly when real events and opportunities for practice are uncommon [1, 2, 3, 4].

This distinction matters because paediatric resuscitation is a low‐frequency, high‐stakes, team‐based activity. Formal renewal courses provide shared language and standards, but maintenance between courses depends on repeated opportunities to practise, receive feedback and return to identified gaps [1, 2, 5, 6, 7, 8]. The practical question for the clinical teacher is therefore not only ‘Is this learner certified?’ but also ‘When did this learner last practise the behaviour that will be needed in the next emergency?’

This toolbox focuses on resuscitation after the immediate newborn period, including infants and older children. Delivery‐room neonatal resuscitation uses distinct algorithms, equipment and certification systems and is outside its primary scope, although evidence from neonatal education helps illustrate how practice needs may vary by skill and learner [4]. The toolbox is intended as a flexible starting point rather than a standardised programme.

### 1.1 From Certification to Maintained Readiness

A readiness approach does not replace life‐support certification. It adds a local maintenance habit between formal courses. Certification is usually documented as completion; readiness is better approached as current performance that can be observed, coached and revisited. A small amount of data can help a unit distinguish attendance from learning: what was practised, what was observed and what should be repeated next.

Table 1 contrasts a certification‐focused approach with a maintained‐readiness approach.

**TABLE 1:** From certification alone to maintained readiness.

Certification alone	Maintained readiness
Course completed at a defined time	Current capability revisited over time
Episodic renewal	Repeated short practice between renewals
Pass or fail	A performance gap followed by another attempt
Individual technical skill	Individual and team behaviours
Attendance or completion recorded	Skill practised, feedback and next step recorded
Confidence presumed	Confidence compared with observed performance

### 1.2 Why These Elements Are Included

Three educational principles support the toolbox. First, distributed practice allows learners to revisit a skill before it becomes unfamiliar. Evidence generally favours repeated practice over reliance on one infrequent course, but the optimal dose and frequency are not settled. In a neonatal ventilation study, different skill elements required different combinations of practice frequency and number of cases, supporting individualised rather than uniform schedules [1, 4, 5, 6, 7, 8].

Second, deliberate practice requires a narrow target and an opportunity to improve it. Rapid‐cycle deliberate practice uses repeated performance and directed feedback before progression. It is associated with better immediate learner performance, although evidence of superiority over traditional simulation and evidence for long‐term or clinical outcomes remain limited [1, 9]. The routine below draws on its practice–feedback–repeat logic. It is not presented as a complete rapid‐cycle deliberate practice curriculum.

Third, feedback should lead to action. Information about rate, depth, mask seal or communication is most useful when the learner can apply it immediately in a second attempt. Brief reflection can then help the learner compare perceived and observed performance and identify a concrete next step. Reflection is more likely to be useful when it is facilitated, connected to evidence and directed towards future action rather than requested as an isolated exercise [10, 11].

**BOX 1 | An illustrative 10‐min readiness booster.**

**Table:** 

Step	Activity	Duration
1	Frame the activity: state one objective, explain that practice is formative, and check whether any adjustment is needed.	1 min
2	First observed attempt using local equipment.	3 min
3	Give focused feedback from device data or direct observation.	2 min
4	Repeat the same task and apply the feedback.	2 min
5	Facilitate one reflection question and agree one next step.	2 min
*Note:* The duration and sequence are pragmatic, not an evidence‐derived minimum or optimum. Adapt the frequency, activities, participants, equipment and pace to local resources, resuscitation systems and learner needs.

### 1.3 Toolbox Item 1: Choose a Small, Adaptable Practice Dose

Ten minutes was selected as a practical local design choice. It is long enough to establish one objective, observe an attempt, provide feedback, repeat the task and identify a next step, yet short enough to attach to orientation, a unit huddle, a teaching session or a skills day. This exact routine has not been prospectively tested and should not be interpreted as a one‐size‐fits‐all dose.

The frequency should also be adapted. A unit may begin monthly, attach practice to an existing educational schedule or repeat a session when simulation or event review identifies a recurring gap. Some learners or skills will need more frequent practice; others may need longer preparation or a different format. Repetition matters more than novelty. A task can return in a spiral of increasing complexity: mechanics first, then speed, communication, role integration and a short clinical context [1, 4, 5].

*Repetition matters more than novelty*.

### 1.4 Toolbox Item 2: Make Performance Visible and Repeat the Task

Familiarity is not the same as performance. Where available, feedback‐enabled manikins can display compression rate, depth, recoil and ventilation measures. Where technology is unavailable, a short checklist and direct observation can focus attention on two or three behaviours. The purpose is not to produce a comprehensive score. It is to make one improvement target visible [1, 5, 6, 7, 8].

*Familiarity is not the same as performance*.

Feedback should be specific and usable: ‘Your rate increased during the handover. On the next attempt, name the incoming compressor and complete the switch within the planned pause’. The learner then repeats the same task. This closes the feedback loop and keeps the session formative rather than punitive [9, 10].

### 1.5 Toolbox Item 3: Facilitate Reflection and Respond to Difficulty

The clinical teacher or designated facilitator should ask one brief question after the second attempt. Useful prompts include ‘What differed between your expected and observed performance?’ and ‘What will you repeat or change next time?’ The facilitator helps translate the answer into one achievable next step and records it only when local practice requires documentation [11].

An important gap should lead to targeted follow‐up, not public remediation. The facilitator can arrange another coached attempt, provide preparatory material or discuss the issue privately. If a learner becomes distressed, the activity should pause. Acknowledge the response, offer a private conversation and connect the learner with appropriate educational or well‐being support. Reflection should never be used to press a learner into disclosing more than is needed for learning.

**BOX 2 | Faculty responsibilities for safety and accessibility.**

**Table:** 

Before practice	Explain the purpose and formative nature of the activity; clarify roles and confidentiality; invite requests for reasonable adjustments; provide advance information when helpful.
During practice	Use respectful, specific language; monitor hierarchy and participation; allow pauses or additional processing time; offer accessible equipment or an alternative way to participate while preserving the learning objective.
After practice	Facilitate one focused reflection; address significant gaps privately; acknowledge distress; arrange follow‐up rather than using an isolated formative performance punitively.

### 1.6 Psychological Safety and Reasonable Adjustments

Psychological safety is not created by declaring a room safe. It is a dynamic perception that speaking, asking for help or discussing an error will not lead to humiliation or punishment. Faculty influence it before, during and after practice through the way they frame the activity, listen, respond to mistakes and repair an interaction that has caused harm [12].

*Psychological safety is not created by declaring a room safe*.

Brief practice should not mean inflexible practice. Learners may need advance preparation, additional processing time, accessible or modified equipment, an observer or coaching role, an alternative physical technique, written reflection or a follow‐up conversation. Reasonable adjustments should be planned with the learner and relevant local support services, preserving the intended learning outcome without assuming that every participant must demonstrate learning in an identical way [13].

### 1.7 Toolbox Item 4: Practise the Paediatric System

Paediatric resuscitation commonly includes respiratory deterioration, bradycardia and airway management as well as chest compressions. A booster can therefore rotate among mask ventilation, airway positioning, compression quality, compressor change, preparation for intubation, leadership and communication. Across sessions, participants should rotate through leadership and other locally relevant roles rather than repeatedly practising only their preferred role.

Team composition should reflect local practice. Ask each participant to state a role, position and first task. Rehearse one handover or closed‐loop instruction. For medication communication, involve the person locally responsible for preparation or confirmation, for example, a nurse, physician, pharmacist or another designated team member. The specific profession will depend on the local team structure; the aim is to make responsibility and communication explicit [14].

Table 2 provides a simple progression for advancing local paediatric resuscitation practice from technical mechanics to team‐based clinical context.

**TABLE 2:** A simple progression for local paediatric resuscitation practice.

Step	Teaching focus	Example activity
1	Mechanics	Compression quality or mask seal with direct observation or device feedback
2	Response to feedback	Repeat the same task and apply one correction
3	Role rotation	Change compressor, airway or leadership role
4	Team communication	Closed‐loop instruction, compressor handover or airway handover
5	Paediatric context	Short respiratory deterioration or bradycardia sequence using local equipment

### 1.8 Toolbox Item 5: Link Practice to Local Learning and Evaluation

A readiness habit needs simple support from the organisation. Units can record the date, skill practised, participating professional groups and one common learning need. This should not become another credentialing burden. Some sessions may document participation or improvement; others should remain purely formative.

Evaluation can remain modest. At programme level, track whether sessions occur and whether relevant professional groups can participate. At learner level, note whether the targeted behaviour improves after feedback or at the next booster. At system level, compare themes from boosters with simulation debriefings, emergency‐event reviews or quality meetings. When a recurring problem appears, use it to select the next practice target.

This toolbox has not been evaluated as a complete intervention, and a 10‐min booster is not designed to demonstrate improved patient outcomes. Its immediate purpose is to create repeated, observed and feedback‐informed practice. Future evaluation should examine feasibility, inclusion, retention, transfer to clinical care and the resources required in different settings.

**BOX 3 | Common pitfalls and practical safeguards.**

**Table:** 

Pitfall	Safeguard
Treating 10 min as a fixed prescription	Adapt duration and frequency to the skill, learner and setting.
Trying to teach the whole algorithm	Choose one observable objective.
Making practice feel punitive	Normalise skill decay and keep most sessions formative.
Using one professional model everywhere	Rehearse the roles and responsibilities used locally.
Equating brief with high pressure	Offer preparation, pauses, alternative participation and follow‐up.
Tracking attendance only	Record the skill, observed gap and next practice target.
Depending on one faculty champion	Attach boosters to an existing recurring educational activity.

## 2 Conclusion

Life‐support certification remains necessary, but it is a point‐in‐time achievement rather than a complete measure of current readiness. Clinical teachers can support maintenance through brief, repeated and observable practice that links feedback to another attempt, includes facilitated reflection and rehearses the local team system.

The 10‐min sequence offered here is one practical way to begin, not a universal standard. Its value lies in being small enough to repeat and flexible enough to adapt. The goal is not another certificate. It is a local habit of noticing what is needed, practising it and returning to it before the next child requires resuscitation.

*It is a local habit of noticing what is needed, practising it and returning to it before the next child requires resuscitation*.

## Author Contributions

**Weerapong Lilitwat:** conceptualisation, writing – original draft, writing – review and editing, formal analysis, project administration, resources, visualisation.

## Funding

The author has nothing to report.

## Ethics Statement

This Clinical Teacher's Toolbox article does not report research involving human participants, identifiable participant data or animal data.

## Consent

The author has nothing to report.

## Conflicts of Interest

The author declares no conflicts of interest.

## Data Availability

No new data were generated or analysed for this manuscript.
